# Plasma circN4BP2L2 is a promising novel diagnostic biomarker for epithelial ovarian cancer

**DOI:** 10.1186/s12885-021-09073-z

**Published:** 2022-01-03

**Authors:** Li Ning, Jinghe Lang, Lingying Wu

**Affiliations:** 1 Department of Gynecologic Oncology, National Cancer Center/National Clinical Research Center for Cancer/Cancer Hospital, Chinese Academy of Medical Sciences and Peking Union Medical College, Beijing, 100021 China; 2 Department of Gynecology and Obstetrics, Peking Union Medical College Hospital, Chinese Academy of Medical Sciences and Peking Union Medical College, Beijing, 100730 China

**Keywords:** Epithelial ovarian cancer, Circular RNA, Human, Biological markers, Diagnosis

## Abstract

**Background:**

Circular RNAs (circRNAs) are more stable than linear RNA molecules, which makes them promising diagnostic biomarkers for diseases. By circRNA-sequencing analysis, we previously found that circN4BP2L2 was significantly decreased in epithelial ovarian cancer (EOC) tissues, and was predictive of disease progression. The aim of this study was to evaluate the diagnostic value of plasma circN4BP2L2 in EOC.

**Methods:**

Three hundred seventy-eight plasma samples were acquired prior to surgery. Samples were obtained from 126 EOC patients, 126 benign ovarian cyst patients, and 126 healthy volunteers. CircN4BP2L2 was assessed using reverse transcription-quantitative polymerase chain reaction (RT-qPCR). Cancer antigen 125 (CA125) and human epididymis protein 4 (HE4) were assessed using enzyme-linked immunosorbent assay (ELISA). EOC cells were transfected with small interference RNAs (siRNAs) and cell proliferation, migration, invasion, cell cycle and cell apoptosis were performed to assess the effect of circN4BP2L2 in EOC. Receiver operating curve (ROC), the area under the curve (AUC), sensitivity and specificity were estimated.

**Results:**

Plasma circN4BP2L2 was significantly downregulated in EOC patients. Decreased circN4BP2L2 was significantly associated with advanced tumor stage, worse histological grade, lymph node metastasis and distant metastasis in EOC. CircN4BP2L2 inhibited tumor cell migration and invasion in vitro. CircN4BP2L2 could significantly separate EOC from benign (AUC = 0.82, *P* <  0.01) or normal (AUC = 0.90, *P* <  0.01) cohort. Early stage EOC vs benign (AUC = 0.81, *P* <  0.01) or normal (AUC = 0.90, *P* <  0.01) cohort could also be distinguished by circN4BP2L2. In discrimination between EOC cohort and benign or normal cohort, circN4BP2L2 performed equally well in both pre- and post-menopausal women. The combination of circN4BP2L2, CA125 and HE4 showed high sensitivity and specificity in detecting EOC cases.

**Conclusions:**

Plasma circN4BP2L2 is significantly downregulated in EOC and might serve as a promising novel diagnostic biomarker for EOC patients, especially in early stage EOC cases. CircN4BP2L2 might act as an adjunct to CA125 and HE4 in detecting EOC. Further large-scale studies are warranted to verify our results.

## Background

Circular RNA (circRNA) is a novel subtype of non-coding RNAs with variant length ranging from hundred to thousand nucleotides [1, 2]. They are produced from back-splicing events in precursor mRNAs, and can be transcribed by RNA polymerase II as efficiently as linear RNAs [3, 4]. Despite their first discovery in 1976, circRNAs had been regarded as splicing errors until recent [5]. The rapid advancement in next-generation sequencing technology and bioinformatic approaches have identified numerous endogenous circRNAs [6].

Unlike linear RNAs with 5′ and 3′ ends, the covalently closed loop structures of circRNAs make them more stable against Ribonuclease R [7]. Besides, circRNAs are abundantly and widely present in eukaryotic cells [8]. Despite a few exceptions, most circRNAs are highly conserved among various species [9]. Moreover, the expression of circRNAs is spatial-temporal and cell-type specific [10]. These characteristics of circRNAs make them promising biomarkers for disease detection [11].

Accordingly, a body of studies have been performed to investigate the diagnostic value of circRNAs in various diseases. Zhang et al. [12] found that upregulated serum circ_0068481 could be a novel biomarker for disease diagnosing and outcome prediction in patients with idiopathic pulmonary arterial hypertension. Zhu et al. [13] reported that upregulated serum circ_0000885 might serve as a diagnostic biomarker for osteosarcoma. Lin et al. [14] identified a panel of three plasma circRNAs (circ-CCDC66, circ-ABCC1 and circ-STIL) which might act as novel diagnostic biomarkers for colorectal carcinoma. These studies indicated that circRNAs might be promising molecular biomarkers for diagnosing diseases, including cancer.

Epithelial ovarian cancer is the most lethal malignancy in female genital tract [15, 16]. Due to lacking of sensitive detection method, most patients are diagnosed at advanced stages, resulting in a dismal 5-year survival rate of about 30–40% [17]. Cancer antigen 125 (CA125) and human epididymis protein 4 (HE4) are two FDA-approved biomarkers for EOC [18]; however, both of them are limited to their relatively low sensitivity [19, 20]. To improve the prognosis of EOC patients, it is imperative to identify novel sensitive biomarkers for early detection of EOC. Efforts have been made to investigate the role of circRNAs in EOC diagnosis. We previously found that serum circBNC2 was significantly downregulated in EOC patients, and might serve as a promising biomarker for early detection of EOC [21]. Wang et al. [22] also reported that upregulated serum circSETDB1 could separate serous ovarian cancer patients from normal volunteers, and was predictive of disease progression. Both studies suggested that circRNAs might help to enhance the diagnostic capacity of EOC; however, more researches with larger sample size are warranted to further clarify the diagnostic value of circRNAs in EOC.

We have previously identified differential circRNA expression profiles in EOC by performing circRNA-sequencing analysis. Particularly, we found that circN4BP2L2 was significantly downregulated in EOC, and was predictive of disease progression [23]. CircN4BP2L2 is spliced from a nuclear protein N4BP2L2, which was firstly identified to be increased following treatment of Jurkat T lymphocytes with the herpesvirus drug phosphonoformate [24]. N4BP2L2 plays an important role in transcriptional regulation, and is found to be critical in neutrophil differentiation [24]. So far, the characterization of circN4BP2L2 in EOC remains largely unknown. In this study, we aimed to investigate the diagnostic value of circN4BP2L2 in EOC.

## Methods

### Study population

A total of 386 women were initially recruited. Eight malignancies were excluded because of non-epithelial ovarian cancer (*n* = 7; 4 juvenile granulosa cell tumor, 1 endodermal sinus tumor, and 2 dysgerminoma) and metastasis from colorectal cancer (*n* = 1). The eligible study population (*n* = 378) consisted of age and menopause-matched women with EOC (*n* = 126), benign ovarian cyst (*n* = 126), and healthy volunteers (*n* = 126). Menopause status was defined as 1 year of amenorrhea in women over 47 years of age. The study protocol was approved by the local ethics committee at the National Cancer Center/National Clinical Research Center for Cancer/Cancer Hospital of Chinese Academy of Medical Sciences (CAMS) and Peking Union Medical College Hospital of CAMS and was conducted in accordance with the Declaration of Helsinki. Written informed consent for taking the venous blood and tissues was obtained from all patients and healthy volunteers.

The inclusion criteria were: pathologically confirmed EOC; patients without preoperative radiotherapy, chemotherapy, or target therapy; and follow-up information. Patients with ovarian borderline tumors were excluded. Patients with any other coexisting malignancies were also excluded. Tumor stage and grade were determined according to the International Federation of Gynecology and Obstetrics (FIGO) classification [25]. The clinicopathologic parameters, including age, menopause, histological subtype, FIGO stage, tumor grade, lymph node metastasis (LNM), and distant metastasis are shown in Table 1. Patients in the benign cohort had endometriosis, serous cystadenoma, mucinous cystadenoma, and mature teratoma (Table 1). The healthy volunteers had no concomitant illness.

**Table 1:** The main clinicopathologic parameters of included women (*N* = 378)

	N (%)
Age, average, range ^a^	55 (30–76)
Menopause ^b^	
Pre-M	198/378 (52%)
Post-M	180/378 (48%)
Epithelial ovarian cancer ^c^	
Histological subtype	
Serous carcinoma	73/126 (58%)
Endometrioid carcinoma	30/126 (24%)
Clear cell carcinoma	16/126 (13%)
Mucinous carcinoma	7/126 (5%)
FIGO stage	
I	21/126 (17%)
II	15/126 (12%)
III	82/126 (65%)
IV	8/126 (6%)
Tumor grade	
G1	46/126 (37%)
G3	80/126 (63%)
Lymph node metastasis ^d^	
Yes	50/112 (45%)
No	62/112 (55%)
Distant metastasis	
Yes	56/126 (44%)
No	70/126 (56%)
Benign ovarian cyst ^e^	
Endometriosis	81/126 (64%)
Serous cystadenoma	17/126 (14%)
Mucinous cystadenoma	20/126 (16%)
Mature teratoma	8/126 (6%)

Notes: ^a^ Average age for 378 age-matched included women

^b^Menopause status for 378 menopause-matched included women

^c^The clinicopathologic parameters of patients with epithelial ovarian cancer (*n* = 126)

^d^The information of lymph node metastasis was only available in 112 EOC patients

^e^Histological subtype of patients with benign ovarian cyst (*n* = 126)

*Abbreviations*: *N* number, *M* menopause, *FIGO* International Federation of Gynecology and Obstetrics, *G* grade

### Sample collection

Patients were consecutively and prospectively recruited when admitted for surgery for a clinically suspicious malignant or benign ovarian cyst at the Department of Gynecologic Oncology in Cancer Institution & Hospital of CAMS and Peking Union Medical College Hospital of CAMS, between December 2015 and April 2021. Peripheral venous blood samples were obtained on the surgery day and immediately centrifuged at 3000 rotations per minute for 5 min. Plasma samples were aliquoted and subsequently stored in RNA later at − 80 °C until use.

Moreover, 126 cancer specimens from eligible EOC patients and 80 normal ovarian tissues were collected for circRNA validation by reverse transcription-quantitative polymerase chain reaction (RT-qPCR). EOC specimens were collected from patients who had histologically proven to suffer from EOC and had received cytoreductive surgery or wedge biopsy of ovaries. Normal ovarian tissues were collected from patients who received adnexectomy due to myoma of uterus. EOC specimens were collected from primary sites, and normal ovarian tissues were collected from the surface epithelium. Fresh tissues were collected during surgery, frozen in liquid nitrogen within 5 min following resection, and stored at − 80 °C until use. All the EOC specimens and normal ovarian tissues were confirmed by two independent experienced pathologists.

### Cell culture and transfection

The human EOC cell lines SKOV3, OVCAR3, CAOV3, HO8910, and TOV-112D, and the human normal ovarian epithelial cell line IOSE80 were purchased from the Type Culture Collection of the Chinese Academy of Sciences (Shanghai, China). SKOV3, OVCAR3, CAOV3, HO8910, TOV-112D, and IOSE80 cells were cultured in DMEM and supplemented with 10% fetal bovine serum (FBS) (BI, Israel); and 1% penicillin/streptomycin (Gibco, USA) in a humidified atmosphere of 5% CO_2_ at 37 °C. Small interference RNAs (SiRNAs) specific to circN4BP2L2 was generated by GenePharma (GenePharma Corporation, Shanghai, China) and was transfected with Lipofectamine 2000 (Invitrogen) according to the manufacturer’s instructions.

### RNA preparation, quality assessment, and RT-qPCR

TRIzol reagent (Takara Bio, Nojihigashi, Kusatsu, Japan) was used to extract total RNA from 200 μL of plasma samples according to the manufacturer’s instructions. NanoDrop 1000 spectro- photometers were used to measure RNA concentration. RNA was set at an OD A260/230 ratio > 1.8 and an OD A260/280 ratio between 1.8 and 2.1. PrimeScript™ RT reagent Kit with gDNA Eraser (Takara Bio, Nojihigashi, Kusatsu, Japan) and SYBR® Premix Ex Taq™ II (Tli RNaseHPlus) (Takara Bio, Nojihigashi, Kusatsu, Japan) were used to perform RT-qPCR according to the manufacturer’s instructions. We used GAPDH as an internal reference gene. The RT-qPCR protocol included a denaturation step (95 °C for 30 s) and 40 cycles of denaturation (95 °C for 5 s) and annealing (60 °C for 40 s). The 2^−ΔΔCT^ method was used to calculate the relative expression levels. The primer sequences were as follows: circN4BP2L2 (forward, 5′-CATGGTGTGTCTCGAAAGAAG-3′ and reverse, 5′-CTGTACCCATC TTGATGGTGA-3′) and GAPDH (forward, 5′-AACGTGTCAGTGGTGGACCTG-3′ and reverse, 5′-GAGACCACCTGGTGCTCAGTG-3′).

### Elisa

Plasma CA125 concentrations (Quantikine Human CA125 Immunoassay; R&D Systems, Minneapolis, USA) and plasma HE4 levels (Quantikine Human HE4 Immunoassay; R&D Systems, Minneapolis, USA) were measured using ELISA analyses on plasma according to the manufacturer’s instructions. The assays were conducted on coded samples.

### Cell proliferation assay

For cell proliferation assay, the transfected cells were seeded into 96-well plates at a density of 2000 cells per well. At 0, 24, 48, 72 and 96 h after seeding, cell viability was measured by the cell counting kit-8 (CCK-8) system (Beyotime, China) according to the manufacturer’s instructions. Briefly, each well was added with 10 μl CCK- 8 solution, and the plate was then incubated at 37 °C for 1 h in dark. Absorbance at 460 nm of each well was measured using a microplate reader (Tecan, Switzerland).

### Cloning formation assay

For colony formation assay, the transfected cells were seeded into 6-well plates at a density of 6000 cells per well and maintained in DMEM medium containing 10% FBS. After 8 days, the cells were fixed with methanol and staining with 0.1% crystal violet, and the colonies were then imaged and counted.

### Migration and invasion assays

For migration and invasion assays, about 1 × 10^5^ transfected cells were suspended in 200 μl of serum-free medium and seeded into the upper chambers of each transwell (8 μm pore size, Costar), which was coated with or without Matrigel (BD Biosciences, USA) for the migration and invasion assays. Medium containing 10% FBS was added to the bottom chamber as a chemoattractant. The cells were incubated at 37 °C with 5% CO_2_ for 24 h for the migration and invasion assays. After incubation, cells in the top chamber were removed with cotton swabs and the cells on the lower surface were fixed with methanol, stained with 0.1% crystal violet, and photographed under a microscope at × 100 magnification (Olympus, Japan).

### Cell cycle and apoptosis assays

For cell cycle assay, the transfected cells were stained with propidium iodide by the cell cycle and apoptosis analysis Kit (Beyotime, China) and then measured by flow cytometry (BD Accuri C6, USA). The ratios of cells in the G0/G1, S, G2 phases were counted and compared. To detect cell apoptosis, cells were stained using an annexin V-FITC apoptosis kit (Beyotime, China) and analyzed using flow cytometry. The ratio of early apoptotic cells to late apoptotic cells was compared to the values obtained for the controls in each experiment.

### Statistical analysis

Statistical analyses were conducted using SPSS 24.0 (SPSS Inc., Chicago, IL, USA). The unpaired t test or student’s t test (normal distribution data) or Mann–Whitney test (abnormal distribution data) was used to compare the statistical differences between two groups. Cut-off value for circN4BP2L2 was calculated using Youden index (specificity + sensitivity-1). Cut-off for CA125 < 35 U/mL and for HE4 < 55.86 pmol/L were used. Cases with marker levels below (circN4BP2L2) or above (CA125 and HE4) threshold levels were considered to be positive. The receiver operating characteristic (ROC) curves were constructed to predict the probabilities for each marker and their combinations. The area under the curve (AUC) values were calculated. Sensitivity (sen) and specificity (spe) were calculated for individual markers and their combinations. A value of *P* <  0.05 was considered to be statistically significant for all comparisons.

## Results

### Patient material

Of 378 women eligible for analysis, 126 had EOC, 126 had benign ovarian cyst, and 126 were healthy volunteers. The average age was 55 (range, 30 to 76) years old. Nearly half of the women (180/378, 48%) were post-menopausal. For patients with EOC, the histological subtype included serous (73/126, 58%), endometrioid (30/126, 24%), clear cell (16/126, 13%), and mucinous (7/126, 5%) carcinomas. Most patients were diagnosed at advanced stages (FIGO III + IV: 90/126, 71%). The benign cohort included endometriosis (81/126, 64%), serous cystadenoma (17/126, 13%), mucinous cystadenoma (20/126, 16%), and mature teratoma (8/126, 7%) (Table 1).

### Correlation between circN4BP2L2 and clinicopathologic parameters of EOC

**Fig. 1:**
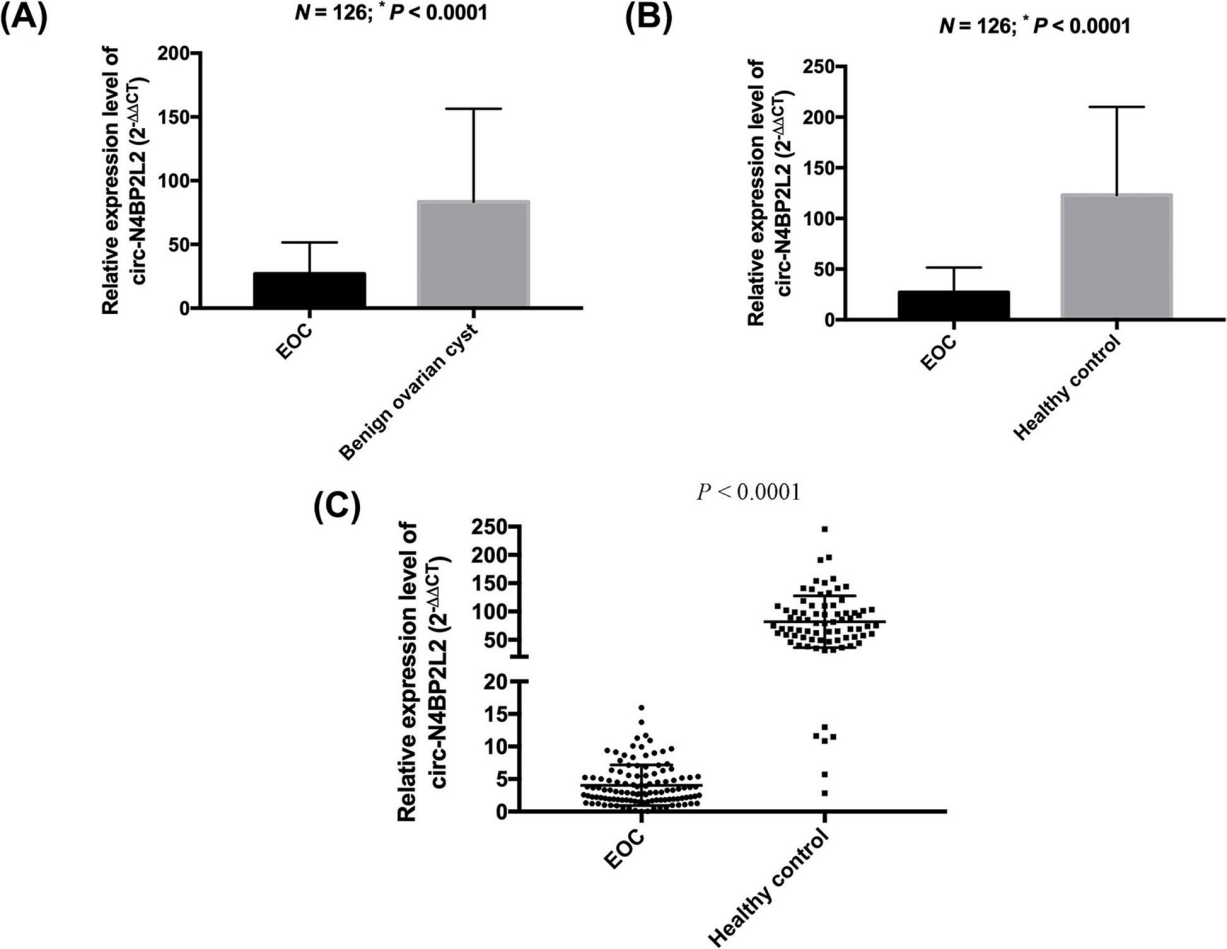
Relative expression level of plasma circN4BP2L2 in EOC (*n* = 126) compared to those in benign ovarian cysts (n = 126) (**A**) and normal controls (*n* = 126) (**B**); and relative expression level of circN4BP2L2 in EOC specimens (*n* = 126) compared to those in normal ovarian tissues (*n* = 80) (**C**)

A total of 126 EOC patients were included to evaluate the correlation between circN4BP2L2 and clinicopathologic parameters of EOC. The expression level of plasma circN4BP2L2 was significantly downregulated in EOC cohort compared to those in benign (*P* <  0.0001) (Fig. 1A) and normal (*P* <  0.0001) (Fig. 1B) cohorts. Besides, the expression level of circN4BP2L2 was significantly decreased in EOC specimens compared to that in normal ovarian tissues (*P* <  0.0001) (Fig. 1C). Notably, we found that decreased plasma circN4BP2L2 was significantly associated with advanced tumor stage (*P* = 0.04), worse tumor grade (*P* = 0.01), lymph node metastasis (*P* = 0.04), and distant metastasis (*P* = 0.03). Nevertheless, the expression level of plasma circN4BP2L2 showed no differences in the context of age and histological subtype of EOC patients (Table 2).

**Table 2:** Correlation between plasma circN4BP2L2 expression level and clinicopathologic parameters of EOC (*N* = 126)

	N (%)	CircN4BP2L2, Mean ± SD	*P*-value
Age, years old			
≤ 50	44/126 (35%)	25.67 ± 13.31	0.06
> 50	82/126 (65%)	29.85 ± 27.64	
Histological subtype			
Serous	73/126 (58%)	22.33 ± 15.57	0.16
Others	53/126 (42%)	24.08 ± 24.79	
FIGO stage			
I-II	36/126 (29%)	28.57 ± 15.92	0.04 ^*^
III-IV	90/126 (71%)	18.94 ± 25.89	
Tumor grade			
G1	46/126 (37%)	41.81 ± 29.98	0.01 ^*^
G3	80/126 (63%)	18.93 ± 13.75	
Lymph node metastasis ^a^			
Yes	50/112 (45%)	18.27 ± 7.41	0.04 ^*^
No	62/112 (55%)	36.24 ± 27.69	
Distant metastasis			
Yes	56/126 (44%)	16.14 ± 8.50	0.03 ^*^
No	70/126 (56%)	34.68 ± 28.91	

Notes: ^a^ The information for lymph node metastasis was only available in 112 EOC patients

*Abbreviations*: *N* number, *FIGO* International Federation of Gynecology and Obstetrics, *G* grade, *SD* standard deviation

### Decreased circN4BP2L2 promoted the progression of EOC cells in vitro

**Fig. 2:**
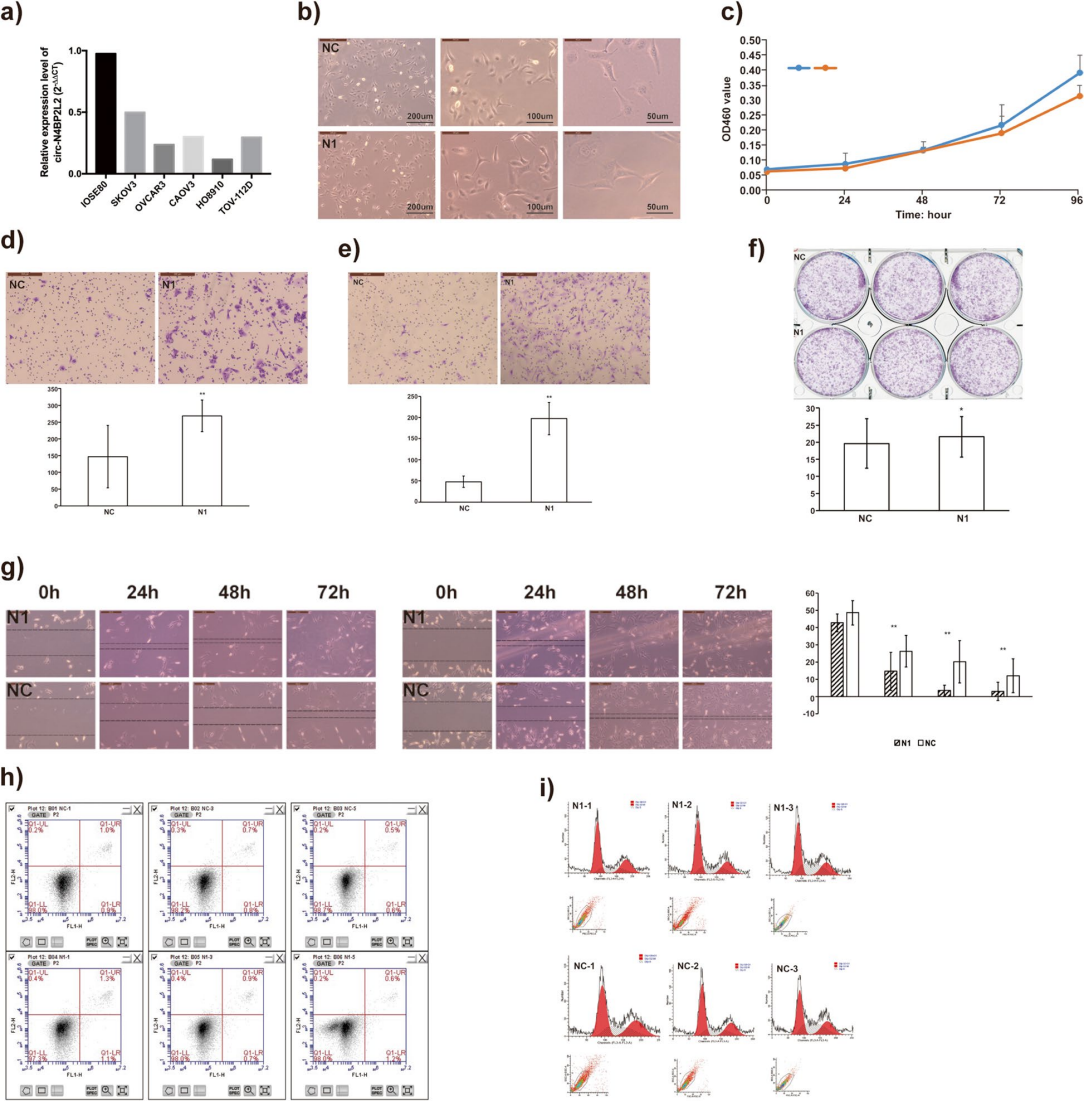
Downregulation of circN4BP2L2 promoted epithelial ovarian cancer cell migration and invasion in vitro. **a** Relative expression level of circN4BP2L2 in 5 different EOC cell lines (SKOV3, OVCAR3, CAOV3, HO8910 and TOV-112D) and one normal ovarian epithelial cell line (IOSE80); **b** After circN4BP2L2 RNA interference, the morphology of tumor cells in interference group (N1) significantly changed into spindle shape compared to that in control group (NC); **c** CircN4BP2L2 expression level did not affect tumor cell proliferation as indicated by CCk-8 assays in SKOV3 cells. Data are mean ± standard deviation from triplicate experiments (*P* > 0.05, Student’s t-test). **d** Transwell migration assay was measured and the results showed that downregulation of circN4BP2L2 promoted tumor cell migration (*P < 0.05, **P < 0.01, Student’s t-test); **e** Transwell invasion assay was measured and the results showed that downregulation of circN4BP2L2 promoted tumor cell invasion (*P < 0.05, **P < 0.01, Student’s t-test); **f** colony formation assays were performed and the results from triplicate experiments demonstrated that circN4BP2L2 did not affect the colony formation of SKOV3 cells (*P < 0.05, **P < 0.01, Student’s t-test); **g** Wound healing assay showed that downregulation of circN4BP2L2 resulted in a faster closing of scratch wounds (*P < 0.05, **P < 0.01, Student’s t-test); **h** and **i** CircN4BP2L2 expression level did not affect tumor cell cycle or apoptosis

Given that circN4BP2L2 was significantly downregulated in EOC tissues and plasmas in our study, we further investigated its potential functional role in EOC cell lines. Firstly, RT-qPCR was used to detect the relative expression level of circN4BP2L2 in five difference EOC cell lines (SKOV3, OVCAR3, CAOV3, HO8910, and TOV-112D) and one normal ovarian epithelial cell line (IOSE80). Our data revealed that circN4BP2L2 was significantly downregulated in five EOC cell lines compared to normal control (Fig. 2a), which was in accordance with previous results. Besides, our results showed that the expression level of circN4BP2L2 was higher in SKOV3 cell line than those in OVCAR3、CAOV3、HO8910, and TOV-112D cell lines (Fig. 2a). Therefore, we chose SKOV3 cell line to perform subsequent functional assays.

We then silenced circN4BP2L2 in SKOV3 cell line by RNA interference technology and constructed interference group (N1) and control group (NC) to evaluate whether reducing the expression level of circN4BP2L2 could affect tumor cell proliferation, apoptosis, cell cycle, invasion and migration. As shown in Fig. 2b, after RNA interference, the morphology of tumor cells in N1 group significantly changed into spindle shape compared to that in NC group.

Functionally, CCK-8 assays revealed that the viability of SKOV3 was not affected in circN4BP2L2 silencing group compared with that in control group (Fig. 2c). Besides, colony numbers of circN4BP2L2 silencing cells were similar with those of control group (Fig. 2f). Notably, transwell migration and invasion assays indicated that the migration (Fig. 2d) and invasion (Fig. 2e) abilities of SKOV3 cell lines were suppressed by circN4BP2L2 (*P* <  0.05), which was in accordance to the wound healing assays (P <  0.05) (Fig. 2g). Flow cytometry analysis was further performed to evaluate whether circN4BP2L2 could affect EOC cell phenotype by altering the cell cycle profile and apoptosis. As shown in Fig. 2h and i, no more cells were distributed in G1 phase after silencing circN4BP2L2, which suggested that circN4BP2L2 could not induce G1/S cell cycle arrest. In addition, apoptosis assays also revealed that circN4BP2L2 could not induce apoptosis in EOC cells. These data suggested that circN4BP2L2 inhibited the migration and invasion of EOC cells in vitro.

### The diagnostic value of circN4BP2L2, CA125 and HE4 in EOC

**Fig. 3:**
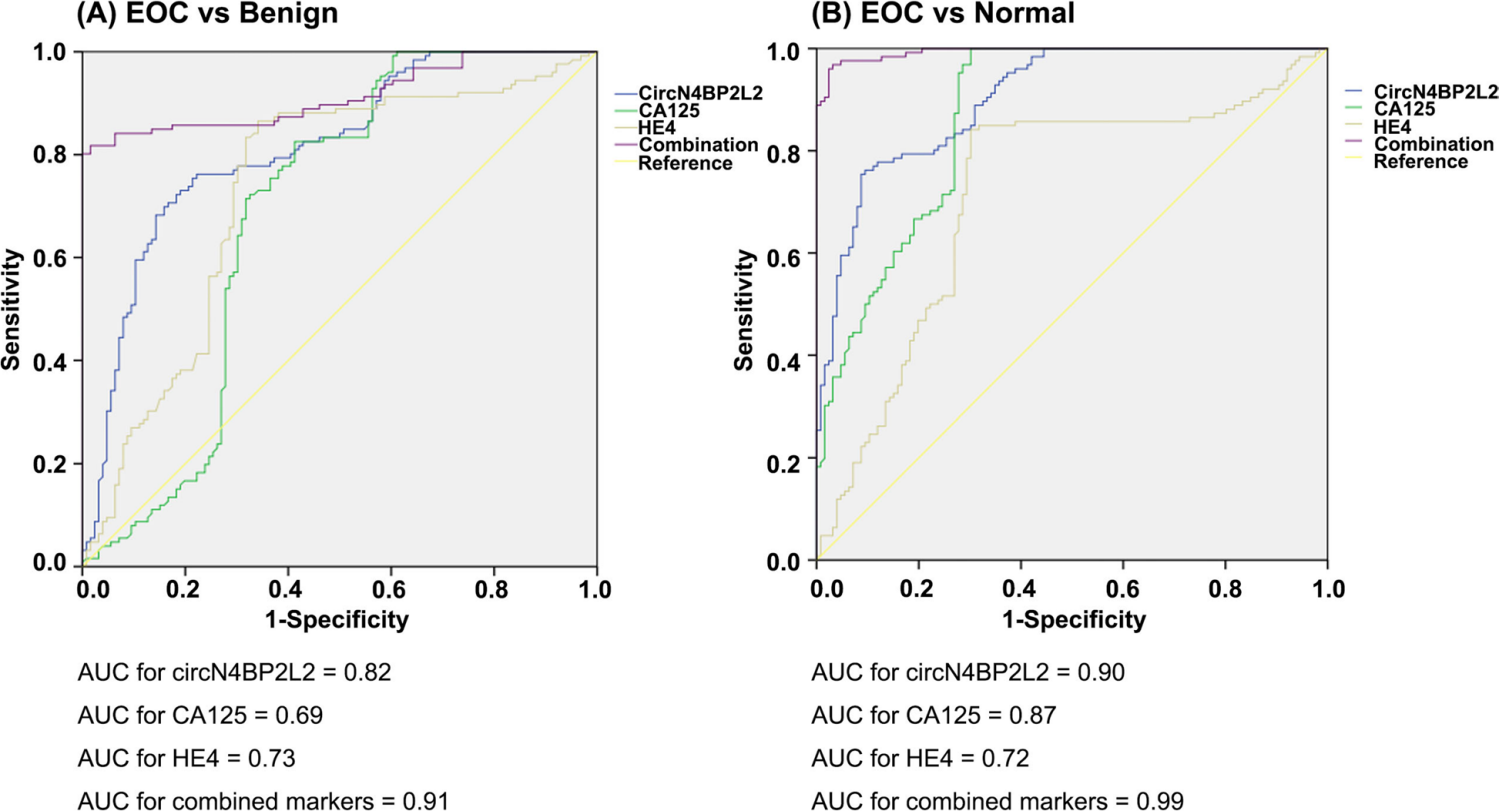
ROC AUC for circN4BP2L2, CA125, HE4 and the combination (circN4BP2L2, CA125, and HE4) in epithelial ovarian cancer compared to those in benign (**A**) and normal (**B**) cohorts

CircN4BP2L2, CA125 and HE4 could significantly separate EOC from benign (*P* <  0.01) (Fig. 3A) and normal (*P* <  0.01) (Fig. 3B) cohorts (Table 3). The median value of CA125 ranged from 24.0 U/mL in normal cohort to 54.1 U/mL in benign cohort and 215.2 U/mL in EOC cohort. The median value in EOC cohort for HE4 was 106.3 pmol/L, which was significantly higher than those in benign (46.1 pmol/L) and normal (45.5 pmol/L) cohorts. The difference was also remarkable for circN4BP2L2, with median value ranged from 95.8 in normal cohort to 62.9 in benign cohort and 17.5 in EOC cohort.

**Table 3:** CircN4BP2L2, CA125, and HE4 expression levels according to histology, menopause status, and FIGO stage; ROC AUC, sensitivity, specificity, and significant difference in EOC cohort vs benign ovarian cyst and normal cohorts

EOC cohort		Benign cohort					Normal cohort				
	Median (range)	Median (range)	ROC AUC (95% CI)	Sen	Spe	*P*-value	Median (range)	ROC AUC (95% CI)	Sen	Spe	*P*
CircN4BP2L2	17.5 (0.5–89.6)	62.9 (1.0–367.2)	0.82 (0.76–0.87)	80%	78%	< 0.01^*^	95.8 (10.9–410.7)	0.90 (0.87–0.94)	82%	90%	< 0.01^*^
Pre-M	23.4 (2.0–89.6)	61.5 (1.0–344.3)	0.80 (0.73–0.88)	79%	77%	< 0.01^*^	87.4 (10.9–389.5)	0.90 (0.85–0.95)	79%	91%	< 0.01^*^
Post-M	12.2 (0.5–77.2)	64.3 (1.9–367.2)	0.83 (0.76–0.90)	72%	87%	< 0.01^*^	109.4 (13.1–410.7)	0.90 (0.85–0.96)	77%	88%	< 0.01^*^
Early stage^a^	12.6 (1–86.3)		0.81 (0.73–0.89)	69%	79%	< 0.01^*^		0.90 (0.85–0.95)	92%	71%	< 0.01^*^
Late stage^a^	17.5 (0.5–89.6)		0.82 (0.76–0.88)	74%	84%	< 0.01^*^		0.91 (0.87–0.95)	79%	91%	< 0.01^*^
CA125	215.2 (16.3–5801)	54.1 (12.7–372.7)	0.69 (0.62–0.76)	73%	24%	< 0.01^*^	24.0 (4.1–68.8)	0.87 (0.83–0.92)	73%	72%	< 0.01^*^
Pre-M	219.7 (16.3–5000)	54.8 (12.7–361.5)	0.69 (0.59–0.78)	74%	24%	< 0.01^*^	25.2 (6.7–68.8)	0.87 (0.81–0.93)	74%	68%	< 0.01^*^
Post-M	202 (16.5–5801)	50.9 (16.3–372.7)	0.68 (0.58–0.79)	72%	23%	< 0.01^*^	22.8 (4.1–67.7)	0.87 (0.81–0.93)	72%	77%	< 0.01^*^
Early stage^a^	28.6 (16.3–143.7)		0.33 (0.22–0.44)	42%	24%	< 0.01^*^		0.68 (0.58–0.78)	42%	72%	< 0.01^*^
Late stage^a^	477.2 (21.8–5801)		0.83 (0.77–0.89)	86%	24%	< 0.01^*^		0.95 (0.92–0.98)	86%	72%	< 0.01^*^
HE4	106.3 (28.3–286.6)	46.1 (12.0–219.0)	0.73 (0.67–0.80)	67%	83%	< 0.01^*^	45.5 (11.2–235.0)	0.72 (0.65–0.78)	67%	85%	< 0.01^*^
Pre-M	79.2 (31.7–218.0)	46.5 (14.0–219.0)	0.70 (0.61–0.79)	54%	80%	< 0.01^*^	45.9 (12.4–235.0)	0.66 (0.57–0.76)	54%	83%	< 0.01^*^
Post-M	114.0 (28.3–286.6)	46.0 (12.0–207.0)	0.86 (0.79–0.93)	82%	87%	< 0.01^*^	43.6 (11.2–201.8)	0.83 (0.75–0.91)	82%	87%	< 0.01^*^
Early stage^a^	70.4 (28.3–207.0)		0.64 (0.53–0.74)	42%	83%	0.01^*^		0.62 (0.52–0.73)	42%	85%	0.02^*^
Late stage^a^	120.3 (31.7–286.6)		0.81 (0.75–0.88)	78%	83%	< 0.01^*^		0.79 (0.73–0.86)	78%	85%	< 0.01^*^
Combination			0.91 (0.88–0.95)	89%	87%	< 0.01^*^		0.99 (0.98–1.00)	91%	96%	< 0.01^*^
Pre-M			0.97 (0.95–0.99)	85%	83%	< 0.01^*^		1.00	90%	95%	< 0.01^*^
Post-M			0.97 (0.94–0.99)	92%	90%	< 0.01^*^		1.00	94%	98%	< 0.01^*^

^a^ According to International Federation of Gynecology and Obstetrics staging

*Abbreviations*: *EOC* epithelial ovarian cancer, *ROC* receiver operating characteristic curve, *AUC* area under curve, *95% CI* 95% confidence interval, *Sen* sensitivity, *Spe* specificity, *Pre-M* Pre-menopause, *Post-M* Post-Menopause

Individually used in discrimination between EOC and benign cohorts, the ROC AUC was highest for circN4BP2L2 (AUC = 0.82), followed by HE4 (AUC = 0.73) and CA125 (AUC = 0.69). CircN4BP2L2 also had higher sensitivity (80%) than CA125 (73%) and HE4 (67%). The specificity of circN4BP2L2 (78%) and HE4 (83%) were higher than that of CA125 (24%).

Individually used in discrimination between EOC and normal cohorts, the ROC AUC for circN4BP2L2 (AUC = 0.90) and CA125 (AUC = 0.87) were higher than that of HE4 (AUC = 0.72). Similarly, circN4BP2L2 had higher sensitivity and specificity (Sen, 82%; Spe, 90%) than HE4 (Sen, 67%; Spe, 85%) and CA125 (Sen, 73%; Spe, 72%).

Contrast to individual biomarker, the combination of all three biomarkers (circN4BP2L2, CA125, and HE4) had higher ROC AUC when comparing EOC with benign (AUC = 0.91) or normal (AUC = 0.99) cohort. The combination also had higher sensitivity and specificity in discrimination between EOC and benign (Sen, 89%; Spe, 87%) or normal (Sen, 91%; Spe, 96%) cohort.

### CircN4BP2L2, CA125 and HE4 evaluation in pre- and post-menopausal EOC

We subsequently investigated the diagnostic value of circN4BP2L2, CA125 and HE4 in pre- and post-menopausal EOC patients.

**Fig. 4:**
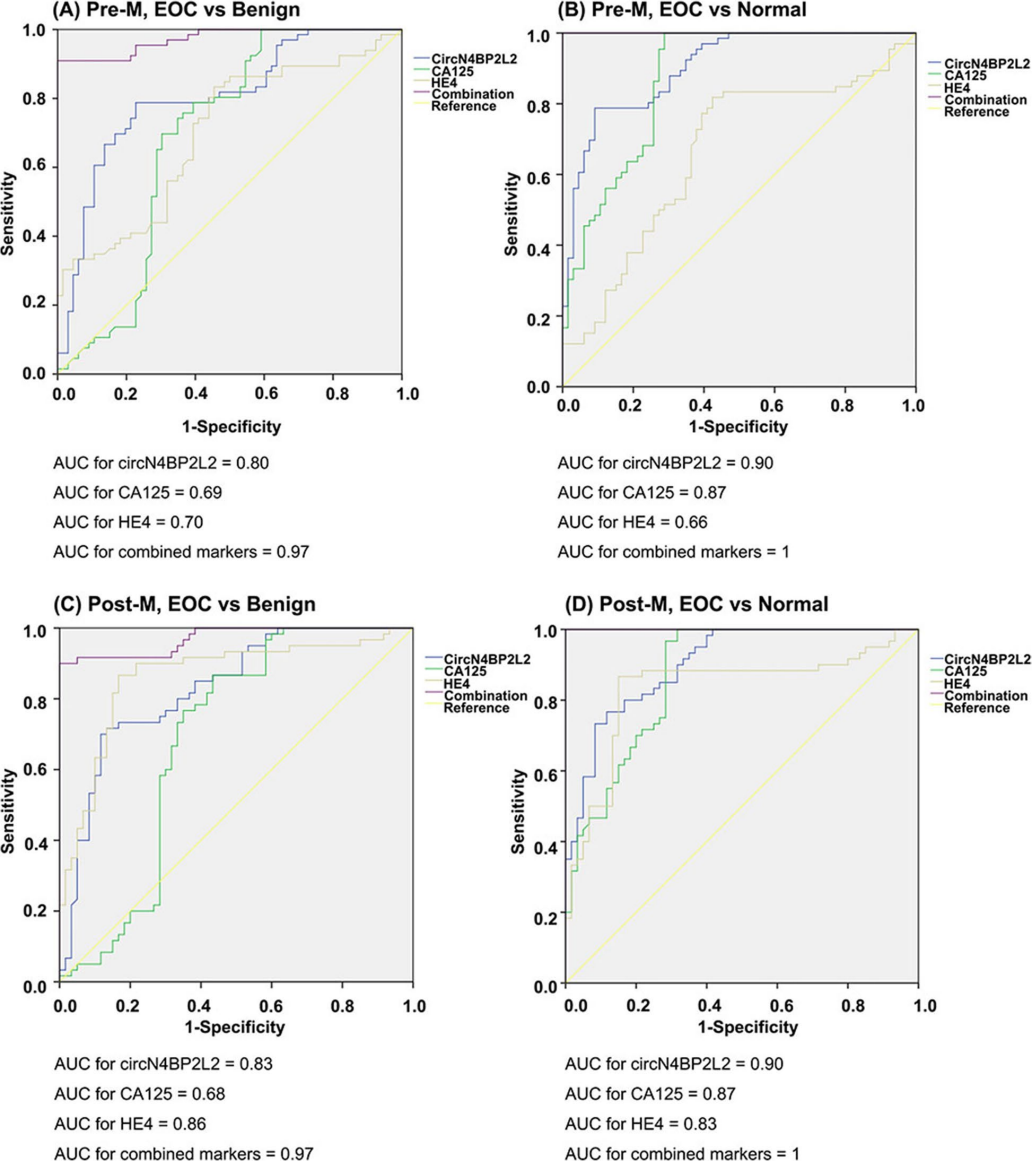
ROC AUC for circN4BP2L2, CA125, HE4 and the combination (circN4BP2L2, CA125, and HE4) in pre-menopausal EOC compared to those in pre-menopausal benign (**A**) and normal (**B**) cohorts. ROC AUC for circN4BP2L2, CA125, HE4 and the combination (circN4BP2L2, CA125, and HE4) in post-menopausal EOC compared to those in post-menopausal benign (**C**) and normal (**D**) cohorts

In pre-menopausal women, circN4BP2L2, CA125 and HE4 could separate EOC from benign (*P* <  0.01) (Fig. 4A) and normal (*P* <  0.01) (Fig. 4B) cohorts (Table 3). Individually used, the ROC AUC was highest for circN4BP2L2 when comparing EOC with benign (AUC = 0.80) and normal (AUC = 0.90) cohorts, followed by CA125 (benign cohort, AUC = 0.69; normal cohort: AUC = 0.87) and HE4 (benign cohort, AUC = 0.70; normal cohort: AUC = 0.66). In discrimination between EOC and benign or normal cohort, the sensitivity of circN4BP2L2 (79, 79%) was higher than those of CA125 (74, 74%) and HE4 (54, 54%); the specificity of circN4BP2L2 (77, 91%) and HE4 (80, 83%) were higher than those of CA125 (24, 68%). Compared to individual biomarker, the combination had higher ROC AUC when comparing EOC with benign (AUC = 0.97) or normal (AUC = 1.00) cohort. The combination also had higher sensitivity and specificity in discrimination between EOC and benign (Sen, 85%; Spe, 83%) or normal (Sen, 90%; Spe, 95%) cohort.

In post-menopausal women, circN4BP2L2, CA125 and HE4 could separate EOC from benign (*P* <  0.01) (Fig. 4C) or normal (*P* <  0.01) (Fig. 4D) cohort (Table 3). Individually used in comparing EOC with benign or normal cohort, the ROC AUC of circN4BP2L2 (benign cohort, AUC = 0.83; normal cohort: AUC = 0.90) and HE4 (benign cohort, AUC = 0.86; normal cohort: AUC = 0.83) were higher than those of CA125 (benign cohort, AUC = 0.68; normal cohort: AUC = 0.87). In discrimination between EOC and benign or normal cohort, the sensitivity and specificity of circN4BP2L2 (benign cohort: Sen 72%, Spe 87%; normal cohort: Sen 77%, Spe 88%) and HE4 (benign cohort: Sen 82%, Spe 87%; normal cohort: Sen 82%, Spe 87%) were higher than those of CA125 (benign cohort: Sen 72%, Spe 23%; normal cohort: Sen 72%, Spe 77%). Contrast to individual biomarker, the combination had higher ROC AUC when comparing EOC with benign (AUC = 0.97) or normal (AUC = 1.00) cohort. The combination also had higher sensitivity and specificity in discrimination between EOC and benign (Sen, 92%; Spe, 90%) or normal (Sen, 94%; Spe, 98%) cohort.

### CircN4BP2L2, CA125 and HE4 evaluation in early and late stage EOC

We then evaluated the diagnostic value of circN4BP2L2, CA125 and HE4 with regard to tumor stage. The EOC cohort was further divided into early stage (FIGO I + II; *n* = 36; 29%) and late stage cohorts (FIGO III + IV; *n* = 90; 71%) (Table 1), and respectively compared to the benign and normal cohorts.

**Fig. 5:**
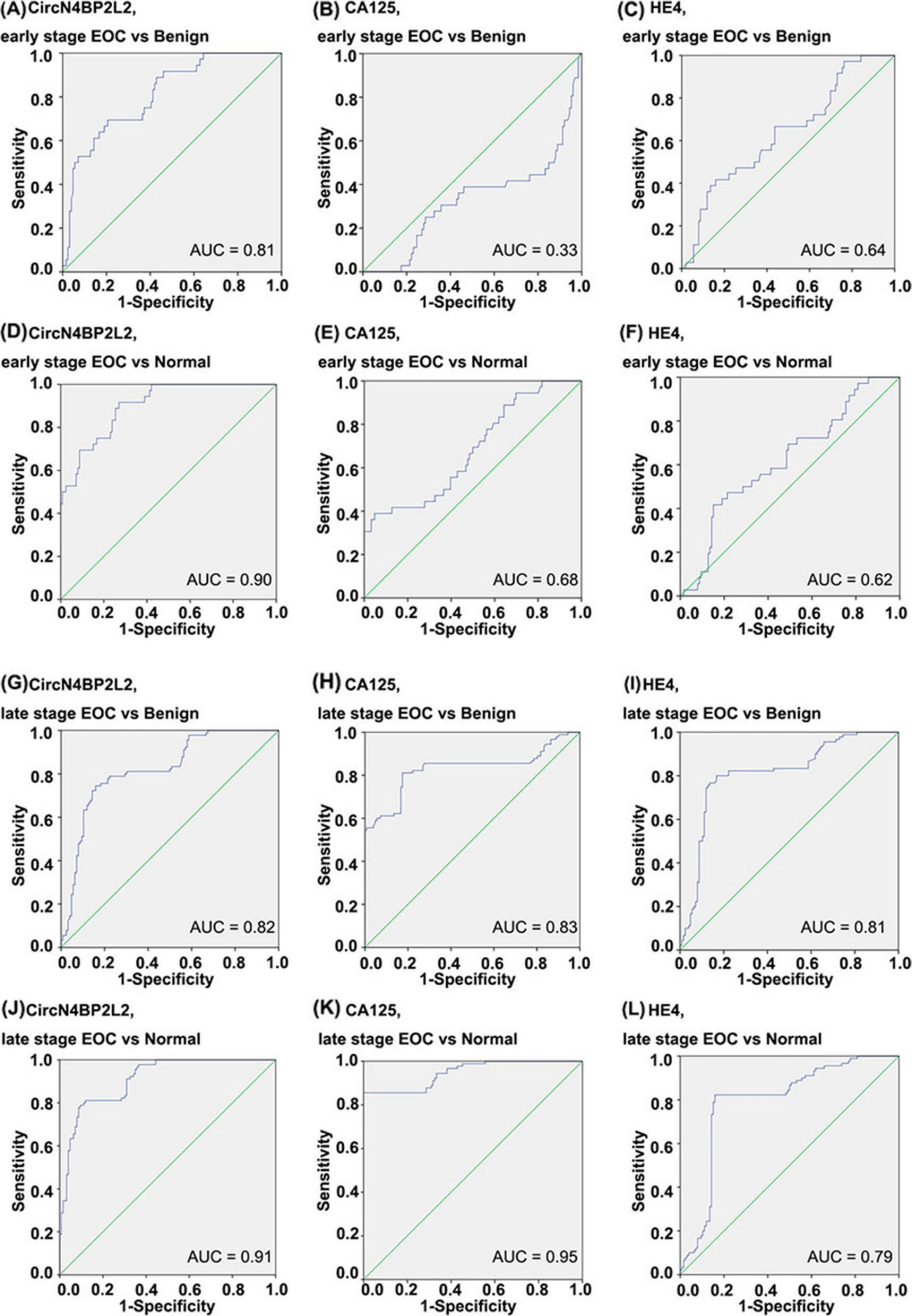
ROC AUC for circN4BP2L2, CA125 and HE4 in EOC with regard to tumor stage. It contains ROC AUC for circN4BP2L2 comparing early stage EOC with benign (**A**) and normal (**D**) cohorts; ROC AUC for CA125 comparing early stage EOC with benign (**B**) and normal (**E**) cohorts; ROC AUC for HE4 comparing early stage EOC with benign (**C**) and normal (**F**) cohorts; ROC AUC for circN4BP2L2 comparing late stage EOC with benign (**G**) and normal (**J**) cohorts; ROC AUC for CA125 comparing late stage EOC with benign (**H**) and normal (**K**) cohorts; ROC AUC for HE4 comparing late stage EOC with benign (**I**) and normal (**L**) cohorts

In discrimination between early stage EOC and benign or normal cohort, statistically significant differences were found between all groups (*P* <  0.01) (Table 3). Individually used in comparing early stage EOC with benign or normal cohort, the ROC AUC was highest for circN4BP2L2 (benign cohort: AUC = 0.81; normal cohort: AUC = 0.90) (Fig. 5, A & D), followed by HE4 (benign cohort: AUC = 0.64; normal cohort: AUC = 0.62) (Fig. 5, C & F), and CA125 (benign cohort: AUC = 0.33; normal cohort: AUC = 0.68) (Fig. 5, B & E). In discrimination between early stage EOC and benign or normal cohort, the sensitivity of circN4BP2L2 (benign cohort: 69%; normal cohort: 92%) were higher than those of HE4 (benign cohort: 42%; normal cohort: 42%) and CA125 (benign cohort: 42%; normal cohort: 42%); the specificity of circN4BP2L2 (benign cohort: 79%; normal cohort: 71%) and HE4 (benign cohort: 83%; normal cohort: 85%) were higher than those of CA125 (benign cohort: 24%; normal cohort: 72%).

When comparing late stage EOC with benign and normal cohorts, statistically significant differences were also obtained between all groups (Table 3). Individually used in discrimination between late stage EOC and benign or normal cohort, the ROC AUC of circN4BP2L2 (benign cohort: AUC = 0.82; normal cohort: AUC = 0.91) (Fig. 5, G & J) and CA125 (benign cohort: AUC = 0.83; normal cohort: AUC = 0.95) (Fig. 5, H & K) were higher than those of HE4 (benign cohort: AUC = 0.81; normal cohort: AUC = 0.79) (Fig. 5, I & L). The sensitivity and specificity of circN4BP2L2 (benign cohort: Sen 74%, Spe 84%; normal cohort: Sen 79%, Spe 91%) and HE4 (benign cohort: Sen 78%, Spe 83%; normal cohort: Sen 78%, Spe 85%) were higher than those of CA125 (benign cohort: Sen 86%, Spe 24%; normal cohort: Sen 86%, Spe 72%).

## Discussion

Nowadays, sensitive biomarkers for EOC diagnosis are scarce. In this study, we found that the expression level of circN4BP2L2 was significantly downregulated in EOC patients. Plasma circN4BP2L2 could separate EOC from benign or normal cohort. Moreover, the combination of three biomarkers (circN4BP2L2, CA125, and HE4) had high sensitivity and specificity in discriminating EOC from benign or normal cohort. These results implied that circN4BP2L2 might serve as a promising diagnostic biomarker for EOC. To the best of our knowledge, we are the first to investigate the diagnostic value of plasma circN4BP2L2 in EOC.

Despite the dismal 5-year survival rate of all EOC cases, it has been reported that patients with stage I disease have an optimistic cure rate of approximately 93% [17, 26]. Therefore, one of the most important strategies to improve the survival outcome of EOC patients is to identify effective method for its early detection [27, 28]. CA125 and HE4 are two FDA-approved biomarkers for diagnosing EOC [29, 30]. However, CA125 is elevated in only 50% of patients with stage I disease [19], and HE4 is also limited by its relatively low sensitivity in diagnosing early stage EOC cases [31]. Transvaginal sonography (TVUS) has been regarded as an important screening tool for early diagnosis of EOC [32]. Nevertheless, TVUS is not preferred since the rate of detected EOC is low, and the survival benefit was not evident when compared with not-screened women [33]. In the UKCTOCS trial enrolling over 50,000 post-menopausal women for annual TVUS, only 45 ovarian cancers were detected, and the mortality of screened women was not found to be reduced over a follow-up of 11 years [34]. In the PLCO cancer screening trial, no difference was obtained in terms of ovarian cancer diagnosis stage, but false positive results were found in approximately 10% of participants, resulting in considerable unnecessary surgeries and corresponding high complication rates [35]. Thus, early detection of EOC should overcome problems of low sensitivity and false positives. In our study with 378 enrolled women, the data showed that circN4BP2L2 could significantly distinguish EOC from benign ovarian cysts (AUC = 0.82; Sen, 80%; Spe, 78%) or normal controls (AUC = 0.90; Sen, 82%; Spe, 90%). Notably, our results revealed that circN4BP2L2 could effectively separate early stage EOC cases from benign (AUC = 0.81; Sen, 69%; Spe, 79%) or normal (AUC = 0.90; Sen, 92%; Spe, 71%) cohort. Additionally, the combination of three biomarkers (circN4BP2L2, CA125, HE4) had showed high sensitivity (benign cohort: 89%, normal cohort: 91%) and specificity (benign cohort: 87%, normal cohort: 96%) in detecting EOC. These results suggested that circN4BP2L2 might be a promising novel biomarker for early detection of EOC patients; and circN4BP2L2 might serve as an adjunct to CA125 and HE4 in detecting EOC, especially in early stage EOC cases. Further large-scale well-designed clinical trials are needed to verify its practicability for clinical application.

By evaluating the correlation between circN4BP2L2 and clinicopathologic parameters of EOC patients, we found that decreased circN4BP2L2 was significantly predictive of advanced tumor stage, worse histological grade, lymph node metastasis and distant metastasis. Accordingly, in our subsequent laboratory research, we demonstrated that low expression of circN4BP2L2 could improve epithelial ovarian cancer cell migration and invasion. These clinical and preclinical analyses highly suggested that circN4BP2L2 might participate in carcinogenesis and development in EOC. However, the function mechanism of circN4BP2L2 is still unknown. Using Arraystar’s homemade miRNA target prediction software, we found that circN4BP2L2 had binding sites for several microRNAs (miRNAs), such as hsa-miR-765, hsa-miR-588, hsa-miR-329-3p, and hsa-miR-135b-5p (available online). Previous studies revealed that these miRNAs were closely related to the development of various malignancies. Zheng et al. [36] reported that hsa-miR-765 could regulate oral squamous cancer cell migration by targeting EMP3. Qian et al. [37] found that hsa-miR-588 could target GRN to regulate cell migration and invasion in lung squamous cell cancer. Li et al. [38] revealed that hsa-miR-329-3p could regulate cell proliferation, migration and invasion by targeting MAPK1 in cervical cancer. Next-generation miRNA sequencing analysis revealed that hsa-miR-135b-5p was dysregulated in gastric cancer tissues [39]. These results suggested that cicN4BP2L2 might participate in the tumorigenesis of EOC by regulating these miRNAs. Further in-depth researches are needed to investigate the function mechanism of circN4BP2L2.

Notably, there has been a long-standing debate regarding whether menopausal status could affect performance of HE4. Our results revealed that HE4 showed better diagnostic performance in post-menopausal ovarian cancer patients. Similar results were also obtained in some previous literatures. In Han et al.’s [30] study, they have enrolled 876 patients with ovarian cysts. Among them, 344 were post-menopausal women (39.3%) and 532 patients were pre-menopausal (60.7%). In detecting ovarian malignancy, the AUC was 0.732 for HE4 (95% CI = 0.692–0.769) in pre-menopausal women; the AUC was 0.845 for HE4 (95% CI = 0.803–0.882) in post-menopausal women. Han et al.’s [30] results showed that HE4 showed better diagnostic capacity in post-menopausal women than that in pre-menopausal women. In Kim et al’s [40] study with 832 ovarian cancer patients, the sensitivity and specificity of HE4 in predicting ovarian cancer were 0.359 and 0.951, respectively, in pre-menopausal patients and 0.718 and 0.952 in post-menopausal patients. Kim et al’s [40] results showed that HE4 showed better sensitivity in diagnosing ovarian cancer in post-menopausal women than that in pre-menopausal women. Likely, Zhang et al.’s [41], Hasanbegovic et al.’s [42], Hada et al.’s [43] and Kristjansdottir et al.’s [44] studies also reported that HE4 had better performance in the diagnosis of post-menopausal ovarian cancer than that of pre-menopausal ovarian cancer. However, other researchers reported that the performance was not affected by menopausal status. In Wei et al’s [45] study of 158 individuals, the sensitivity and specificity of HE4 in predicting ovarian cancer were 78.38 and 70.37%, respectively, in post-menopausal patients and 96.97 and 98.36% in pre-menopausal patients. Wei et al.’s [45] results revealed that HE4 performed alike in both pre- and post-menopausal women in predicting ovarian cancer. Similar result has also been obtained in Terlikowska et al.’s [46] study. In summary, whether menopausal status could affect performance of HE4 are still controversial, which further indicated the limitation of HE4 in diagnosing ovarian cancer.

In this study containing 378 women, it’s worth noting that the CA125 showed limited diagnostic value in distinguishing EOC from benign ovarian cysts. The most likely explanation is that the majority of patients in benign cohort had endometriosis (81/126, 64%). Endometriosis is a painful illness in which the endometrial glands and stroma that normally lines the inside of the uterus, grows and infiltrates outside the uterus [47]. Previous studies have reported that CA125 is highly expressed in endometriosis patients. In a meta-analysis including 22 studies and 3636 participants, CA125 was found to be elevated in approximately half of the patients with endometriosis [48]. Besides, CA125 was not able to detect early stage EOC diseases, which was in accordance with previous studies [40]. These data in turn verified that CA125 was limited in diagnosing EOC [49].

Likely, a body of studies have also been conducted to explore the role of circRNAs in the diagnosis of various cancers. Yin et al. [50] reported that dysregulated plasma hsa_circ_0001785 had better diagnostic accuracy (AUC = 0.784) than CEA (AUC = 0.562) and CA15–3 (AUC = 0.629) in breast cancer. Zhu et al. [51] found that upregulated plasma hsa_circ_0027089 could discriminate HBV-related hepatocellular carcinoma (HCC) from HBV-related cirrhosis and healthy controls, and might serve as a novel diagnostic biomarker for HBV-related HCC. Wang et al. [52] discovered that hsa_circ_0101119 and hsa_circ_0101996 were significantly upregulated in peripheral whole blood of human cervical squamous cell carcinoma (HCSCC), and the combination of hsa_circ_0101119 and hsa_circ_0101996 could be potential diagnostic biomarkers for HCSCC. In the study of Pan et al. [53], circulating exosomal hsa-circ-0004771 was upregulated in colorectal cancer (CRC) patients and might be a novel promising biomarker for CRC diagnosis. Similar results have also been obtained in the studies regarding the diagnostic value of circRNAs in non-small cell lung cancer [54] and osteosarcoma [55]. These researches further verified the feasibility of circRNAs to serve as potentially effective tumor biomarkers.

This study had some limitations. First of all, the number of our recruited EOC patients was relatively small, which might bring overinterpretation to our data. Future large-scale studies are warranted to further verify these results. Secondly, we failed to perform subgroup analysis regarding tumor histology and grade due to the relatively small sample size. Thirdly, in our study of patients with preoperative benign ovarian cysts or suspicious malignancies, the evaluation of circN4BP2L2 as a true diagnostic biomarker was limited, formal study in a screening cohort of women at risk for EOC is needed.

## Conclusions

Our data demonstrated that plasma circN4BP2L2 could separate EOC from benign ovarian cysts and normal controls. CircN4BP2L2 complement CA125 and HE4 had better sensitivity and specificity in distinguishing EOC from benign and normal cohorts. Plasma circN4BP2L2 might serve as a novel biomarker for EOC diagnosis. Further large-scale studies are needed to verify our results.

## Data Availability

The datasets used and/or analyzed during the current study are available from the corresponding author on reasonable request.

## Abbreviations

CircRNAs: Circular RNAs
EOC: Epithelial ovarian cancer
RT-qPCR: Reverse transcription-quantitative polymerase chain reaction
CA125: Cancer antigen 125
HE4: Human epididymis protein 4
ELISA: Enzyme-linked immunosorbent assay
SiRNAs: Small inference RNAs
ROC: Receiver operating curve
AUC: The area under the curve
CAMS: Chinese Academy of Medical Sciences
FIGO: The International Federation of Gynecology and Obstetrics
LNM: Lymph node metastasis
FBS: Fetal bovine serum
NC: Normal control
sen: Sensitivity
spe: Specificity
TVUS: Transvaginal sonography
HCC: Hepatocellular carcinoma
HCSCC: Human cervical squamous cell carcinoma
CRC: Colorectal cancer
